# Measurement of pregnancy-related anxiety worldwide: a systematic review

**DOI:** 10.1186/s12884-022-04661-8

**Published:** 2022-04-15

**Authors:** Kristin Hadfield, Samuel Akyirem, Luke Sartori, Abdul-Malik Abdul-Latif, Dominic Akaateba, Hamideh Bayrampour, Anna Daly, Kelly Hadfield, Gilbert Abotisem Abiiro

**Affiliations:** 1 Trinity Centre for Global Health, School of Psychology, Trinity College Dublin, Dublin, Ireland; 2 Yale School of Nursing, Yale University, New Haven, USA; 3 Barts and The London School of Medicine and Dentistry, Queen Mary University of London, London, England; 4 Institute of Interdisciplinary Research, University for Development Studies, Tamale, Ghana; 5 Ghana Medical Help, Accra, Ghana; 6 Department of Family Practice, University of British Columbia, Vancouver, Canada; 7 Department of Health Services, Policy, Planning, Management and Economics, School of Public Health, University for Development Studies, Tamale, Ghana; 8 Department of Population and Reproductive Health, School of Public Health, University for Development Studies, Tamale, Ghana

**Keywords:** Pregnancy-related anxiety, Pregnancy, Maternal mental health, Systematic review, Measurement, Cross-cultural, Reliability and validity, Background

## Abstract

**Background:**

The perinatal period is often characterized by specific fear, worry, and anxiety concerning the pregnancy and its outcomes, referred to as pregnancy-related anxiety. Pregnancy-related anxiety is uniquely associated with negative maternal and child health outcomes during pregnancy, at birth, and early childhood; as such, it is increasingly studied. We examined how pregnancy-related anxiety is measured, where measures were developed and validated, and where pregnancy-related anxiety has been assessed. We will use these factors to identify potential issues in measurement of pregnancy-related anxiety and the geographic gaps in this area of research.

**Methods:**

We searched the Africa-Wide, CINAHL, MEDLINE, PsycARTICLES, PsycINFO; PubMed, Scopus, Web of Science Core Collection, SciELO Citation Index, and ERIC databases for studies published at any point up to 01 August 2020 that assessed pregnancy-related anxiety. Search terms included pregnancy-related anxiety, pregnancy-related worry, prenatal anxiety, anxiety during pregnancy, and pregnancy-specific anxiety, among others. Inclusion criteria included: empirical research, published in English, and the inclusion of any assessment of pregnancy-related anxiety in a sample of pregnant women. This review is registered on PROSPERO (CRD42020189938).

**Results:**

The search identified 2904 records; after screening, we retained 352 full-text articles for consideration, ultimately including 269 studies in the review based on the inclusion and exclusion criteria. In total, 39 measures of pregnancy-related anxiety were used in these 269 papers, with 18 used in two or more studies. Less than 20% of the included studies (*n* = 44) reported research conducted in low- and middle-income country contexts. With one exception, all measures of pregnancy-related anxiety used in more than one study were developed in high-income country contexts. Only 13.8% validated the measures for use with a low- or middle-income country population.

**Conclusions:**

Together, these results suggest that pregnancy-related anxiety is being assessed frequently among pregnant people and in many countries, but often using tools that were developed in a context dissimilar to the participants’ context and which have not been validated for the target population. Culturally relevant measures of pregnancy-related anxiety which are developed and validated in low-income countries are urgently needed.

**Supplementary Information:**

The online version contains supplementary material available at 10.1186/s12884-022-04661-8.

## Background

Pregnancy is a vulnerable time for the development of mental illness in women. Although estimates vary, a meta-analysis [1] found that 15.2% of pregnant women are diagnosable with an anxiety disorder. Anxiety during pregnancy is associated with poor maternal health, adverse birth outcomes, and negative behavioural and biological development for children across their lifespan [2–4]. However, measures of generalized anxiety during pregnancy tend to explain only a small proportion of variation in these foetal health and birth outcomes [5]. There appear to be components of anxiety during pregnancy that are better captured by pregnancy-related anxiety, which is unique and distinct from generalized anxieties and depression [2, 6, 7]. Pregnancy-related anxiety pertains to the fear, worry, or apprehension surrounding a woman’s pregnancy, childbirth, health of the infant or foetus, and other pregnancy-specific social and financial issues [5]. It also encompasses the fears and concerns about the woman’s physical appearance during pregnancy and her ability to meet expectations of herself as a parent [8]. Pregnancy-related anxiety has unique associations with health during pregnancy, the course of childbirth, and child and maternal outcomes in the postnatal period [2]. It has been linked to increased maternal mortality [9], preterm labour [10], impaired cognitive function among children [11], low birth weight [12], poor maternal bonding [13], and poorer child health and development [14–16].

These associations have contributed to the increased focus on pregnancy-related anxiety among researchers and practitioners. However, as a construct, pregnancy-related anxiety is multidimensional and the salient aspects of it appear to differ by country and context [5, 17]. Although there is less research conducted on pregnancy-related anxiety in low- and middle-income country contexts, qualitative work suggests differences in the domains of this construct in high-income versus low- and middle-income country contexts [5, 18]. In a concept review, maternal mortality, access to adequate health care, and proximity to a health care facility were concerns mentioned by mothers in low- and middle-income countries but not by mothers in high-income locations. Body image, a loss of the foetus, and aspects of parenting and childcare were sources of pregnancy-related anxiety only in samples from high-income countries [5]. The variation in domains of pregnancy-related anxiety across different contexts suggests that the use of tools originally developed to measure pregnancy-related anxiety in high-income countries may not be a valid and reliable approach to identify pregnancy-related anxiety in low- and middle-income countries as these tools may fail to capture locally relevant components of this anxiety. This hinders the accurate identification of predictors of pregnancy-related anxiety and the development, evaluation, and implementation of appropriate interventions.

While there are reviews of the tools used to measure anxiety in pregnancy in general [19, 20], as well as the measurement of pregnancy-related anxiety [21], no systematic review has examined the validation of these measurement tools in relation to their use across different cultural contexts. The aim of this review is to determine how pregnancy-related anxiety is measured across different countries and contexts.

## Methods

### Protocol and guidance

**Fig. 1:**
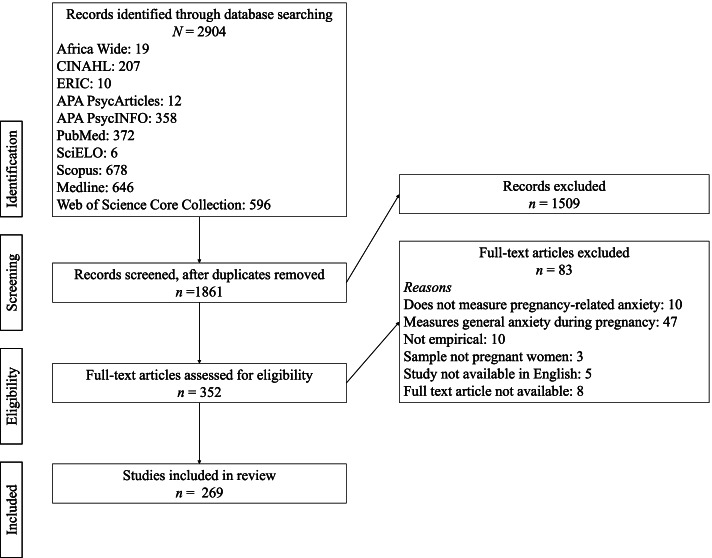
Study selection

This review was prospectively registered on PROSPERO (CRD42020189938) [22]. Each step of the review was informed by Preferred Reporting Items for Systematic Reviews and Meta-Analysis (PRISMA) [23] guidelines, with Fig. 1 showing the data search and refinement in line with those guidelines.

### Search strategy and selection criteria

Author LS searched for relevant academic journal publications discoverable via the following databases: Africa-Wide, CINAHL, MEDLINE, PsycARTICLES, PsycINFO, PubMed, Scopus, Web of Science Core Collection, SciELO Citation Index, and ERIC (Table 1). We conducted all searches on 01 August 2020, including any studies published up to that date. Table 1 includes the full search terms for this review.

**Table 1:** Search terms used, formatted for Pubmed

"pregnancy-related anxiety" [Title/Abstract] or
"pregnancy related anxiety" [Title/Abstract] or
"pregnancy-related worry" [Title/Abstract] or
"pregnancy related worry" [Title/Abstract] or
"prenatal anxiety" [Title/Abstract] or
"perinatal anxiety" [Title/Abstract] or
"prenatal worry" [Title/Abstract] or
"perinatal worry" [Title/Abstract] or
"pregnancy anxiety" [Title/Abstract] or
"pregnancy worry" [Title/Abstract] or
"anxiety during pregnancy" [Title/Abstract] or
"worry during pregnancy" [Title/Abstract] or
"anxiety in pregnancy" [Title/Abstract] or
"worry in pregnancy" [Title/Abstract] or
"pregnancy-specific anxiety" [Title/Abstract] or
"pregnancy specific anxiety" [Title/Abstract] or
"pregnancy-specific worry" [Title/Abstract] or
"pregnancy specific worry" [Title/Abstract]

This shows the formatting of the search terms for Pubmed. Different databases have slightly different formats, but the same search terms were used in each

Studies were eligible if they were original empirical research, published in English, and assessed pregnancy-related anxiety among people who were pregnant. Given conceptual overlap between pregnancy-related anxiety and fear of childbirth, studies which reported measuring solely fear of childbirth among pregnant women were also included. Studies could be either qualitative or quantitative and could be validation studies. We included qualitative and mixed methods studies because we wanted to understand how pregnancy-related anxiety is being assessed: by survey, interview, a combination of methods, or any other way. The centrality of pregnancy-related anxiety to the paper was not an inclusion criterion; studies with a primary focus on another topic but which assessed pregnancy-related anxiety were included. We determined whether a study was conducted in a low-, middle-, or high-income country using the Development Assistance Committee (DAC) list of official development assistance recipients for 2020 [24, 25]. Because multiple authors were involved in title and abstract reviews and in the data extraction and some of the authors solely speak English, papers had to be in English to be included in this review. Full information on the search terms and other search strategy aspects are detailed in the PROSPERO protocol (CRD42020189938) [22] and through the study’s OSF page: https://osf.io/z3vux/.

All 2904 records identified through the database searches were exported to Rayyan, then the 1861 records left after duplicates were removed had the titles and abstracts individually reviewed by at least two authors (KrH, KeH, LS, GAA). Reviewer pairs resolved any discrepancies via consensus discussions.

### Data extraction

Authors KrH, SA, AD, LS, and GAA assessed 352 full-text articles for eligibility, to confirm that they met the inclusion criteria. This was done by two individuals independently for 21.9% of the articles, and otherwise was done by one author. Given that one author (SA) extracted data from the majority of the articles, articles were randomly selected by KrH to be reviewed by an additional author. SA also met weekly with KrH to discuss issues arising and KrH regularly reviewed the extracted data to ensure the quality of this extraction. Any discrepancies in assessments between authors in extracted data were resolved through discussion and then by KrH independently reviewing the article. This ultimately resulted in 269 articles to be included in the systematic review. We then looked at the references sections of included papers to identify potentially relevant papers; this did not identify any additional papers which met the inclusion criteria.

Data from these 269 articles were extracted using a data extraction form developed by KrH and GA. KrH, AD, LS, and GA piloted the chart individually for 10 of the identified studies, comparing results, and then it was used by KrH, SA, AD, LS, and GA to extract data from all included studies. Extracted data included: geographical location, study design, sample (size and specifics), pregnancy-related anxiety measurement (which assessment, language of assessment, evidence of reliability or validity), gestational period when assessed, and identified study limitations. SA then used the COnsensus‐based Standards for the selection of health Measurement INstruments (COSMIN) measures to evaluate each of the validation studies (Supplemental Fig. 1) [26]. COSMIN is an initiative that aims to improve the selection of health outcome measures. In this review, we used the COSMIN checklist, which focuses on the validation of measures of health, to understand how rigorous the validations of new or adapted measures of pregnancy-related anxiety are. Because we were not interested in the quality of the included studies’ findings but rather in how the studies assessed pregnancy-related anxiety, we did not conduct a quality assessment of included articles.

## Results

The study selection is outlined in the PRISMA diagram (Fig. 1). Included studies were published between 2001 and 2020, with 59% published from 2015–2020, suggestive of an increasing focus on this topic. Pregnancy-related anxiety was assessed in studies using a variety of methods and across all trimesters. One-hundred and forty of the studies (52.0%) had a longitudinal or cohort design, 93 (34.6%) were cross-sectional, 17 (6.3%) were experimental (primarily randomized controlled trials or pre- and post-test evaluations), 7 (2.6%) were qualitative studies, 7 (2.6%) were secondary data analyses, 2 (0.7%) were retrospective, 2 (0.7%) were mixed method, and 1 (0.4%) was a case study. Gestational ages studied covered all trimesters, with 135 (50.2%) assessing pregnancy-related anxiety at two or more timepoints during pregnancy. Papers used various methods to assess the ‘levels’ of pregnancy-related anxiety, with some using mean or median splits, others using cut offs (sometimes with limited explanation as to how the cut off was developed), and others providing mean scores and/or ranges.

**Fig. 2:**
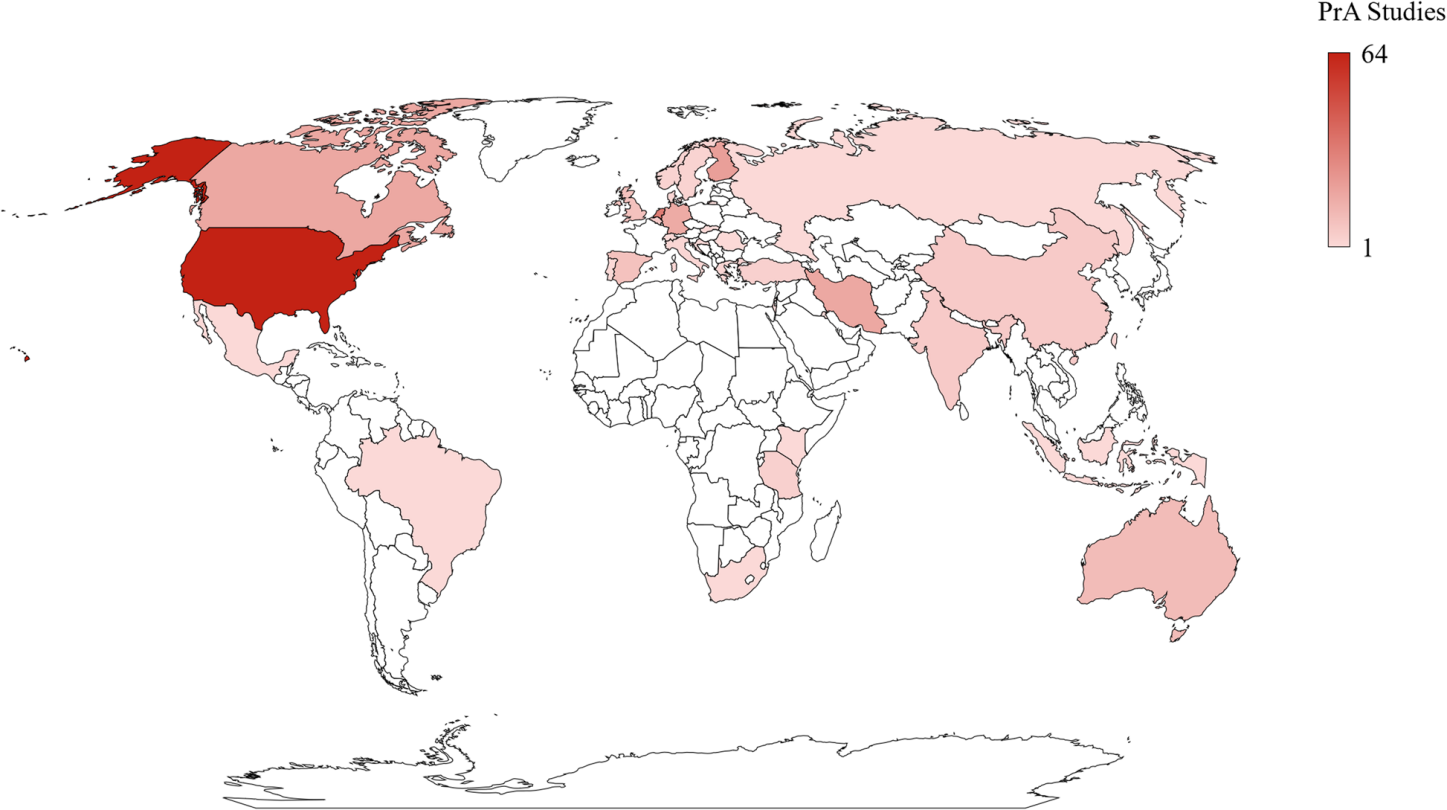
Number of studies on pregnancy-related anxiety by country in which the study was conducted

Included studies were conducted across a large range of countries and contexts, with 210 (78.1%) of the studies conducted in high-income countries, 44 (16.4%) in low- or middle-income countries, and the rest either across multiple countries, online, or unspecified (*n* = 15). Research on pregnancy-related anxiety was conducted in 33 separate countries. The United States was the most frequent location for studies of pregnancy-related anxiety, with 23.8% of the studies conducted there, followed by the Netherlands (12.3%), Finland (7.8%), Iran (6.7%), and Canada (6.7%). Figure 2 shows the number of studies on pregnancy-related anxiety by country.

In total, 19 measures were used in two or more studies; the measures and their usage are described in Table 2. The most commonly used measure in the 269 included studies was the Pregnancy-Related Anxiety Questionnaire (PRAQ) [27]. The PRAQ was frequently used in its original design, although adaptions of it – largely the PRAQ-R [7] and PRAQ-R2 [28] – tended to be used in more recent papers. Following this, the Pregnancy-Related Anxiety Scale (PRAS) [8, 29] was used 39 times and the Cambridge Worry Scale [30] was used 19 times. Twenty-three studies used a one-off measure that was not used in any other published work on assessing pregnancy-related anxiety.

**Table 2:** Measures assessing pregnancy-related anxiety

Tool	Country and language of origin^a^	Number of items	Number of times used?^b^	Other languages / populations validated?	Countries implemented	Languages implemented^c^
Pregnancy-Related Anxiety Questionnaire (PRAQ [27]; PRAQ-R [7] and PRAQ-R2 [28])	Netherlands Dutch	Various, including, original: 34 items, PRAQ-R: 10 items, and PRAQ-R2: 11 items	89	Cantonese, Dutch, English, Finnish, German, Italian, Farsi, Spanish, Swedish, Turkish	Australia, Belgium, China, Finland, Germany, India, Indonesia, Iran, Italy, Netherlands, Norway, Romania, Spain, Switzerland, Tanzania, Turkey, UK, USA	Cantonese, Dutch, English, Farsi, Finnish, German, Hindu, Indonesian, Italian, Mandarin Chinese, Spanish, Swahili, Swedish, Turkish
Pregnancy Related Anxiety Scale [8, 29] (PRAS, sometimes called the Pregnancy-Related Thoughts Scale (PRT) or the Pregnancy-Specific Anxiety Scale (PSA / PSAS) or the Pregnancy Anxiety Scale (PSA)	USA English and Spanish	10-item	39	-	Austria, Canada, Denmark, Germany, India, Iran, Kenya, South Africa, Spain, Tanzania, Taiwan, USA	Danish, German, Kannada, Mandarin Chinese, Swahili
Cambridge Worry Scale [30] (CWS)	UK English	13-, 15- and 16-item versions of the scales were mentioned	19	Farsi, German, Swedish	Austria, Belgium, Canada, Denmark, Germany, Greece, Ireland, Italy, Netherlands, Poland, Portugal, Spain, Sweden, UK	Danish, Dutch, English, French, German, Greek, Italian, Polish, Spanish,
Prenatal Distress Questionnaire [31] (PDQ)	USA English	Mostly the 17-item revised version, although the 9-item and 12-item versions of the scale are included as well	11	English	Canada, Germany, Spain, UK (Northern Ireland), USA	English, German, Spanish
Pregnancy-Specific Anxiety Scale [32] (PSA)	USA English	4-item	10	-	Canada, Netherlands, Russia, UK, USA	English, Russian
Pregnancy Outcome Questionnaire [33] (POQ)	USA English	Two forms – 13-item and 15-item	9	-	Netherlands, UK, USA	English
Wijma Delivery Expectancy / Experience Questionnaire [34] (WDEJ)	Sweden	33 items	8	English, Slovak	Australia, Italy, Norway, Slovakia, Turkey, United Kingdom	Italian, Norwegian, Slovak, Turkish
Fear of Childbirth Questionnaire/Scale [35]	Sweden	19 items (original). Some scales had 11 items	5	-	Finland	-
Pregnancy Anxiety Scale [36] (PAS)	USA English	Various, including 4-, 5-, 6-, 9-, and 10-item scales	5	-	USA	
Childbirth Attitudes Questionnaire [37] (CAQ)	USA	15 items	4	-	Iran, USA	English, Farsi
Pregnancy-Related Anxiety Scale [38–40] (PRAS)	Australia English	Mostly 32 or 33 items, 31 in the Turkish validation	4	Turkish	Australia, Turkey	-
Pregnancy Anxiety Scale [41] (PAS)	USA English	10-item	4	-	Finland, USA	Finnish
Prenatal Social Environment Inventory [42] (PSEI)	USA English	41 items (6 items used to measure PrA)	3	-	USA	-
Pregnancy-Specific Anxiety Questionnaire [43]	China Mandarin Chinese	13 items	3	-	China	-
Lederman Prenatal Self-Evaluation Questionnaire [44] (PSEQ)	USA	53 items	2	-	USA	-
Tilburg Pregnancy Distress Scale [45] (TPDS)	Netherlands Dutch	16 items	2	Dutch, Portuguese	Brazil, Netherlands	Dutch, Portuguese
Perinatal Anxiety Screening Scale [46]	Australia English	31 items	2	-	China, India	-
Pregnancy-Specific Anxiety Inventory [47] (PSAI)	India	40 items	2	-	India	-
Pregnancy Concerns Scale [48] (PCS)	Croatia Croatian	16 items	2	-	Croatia	-
One-off measures	Australia, Canada, Germany, Kuwait, Mexico, Sweden, Turkey, USA	-	23	-	-	-

Some studies included more than one measure of pregnancy-related anxiety

^a^ It was not always possible to tell the language the measure was implemented in

^b^ In the included papers in this systematic review

^c^ In addition to the language in which the measure was initially developed or validated in

Despite the wide use of pregnancy-related anxiety measures across different contexts (Table 2), only two of the measures were developed for use in any low- or middle-income country: the Pregnancy-Specific Anxiety Inventory in India [47] and the Pregnancy-Specific Anxiety Questionnaire in China [43]. Indeed, of the 19 measures used in more than one paper, 9 were developed in the United States and 6 in Europe. Some were validated for use in other populations: the Pregnancy-Related Anxiety Questionnaire [26], Cambridge Worry Scale [30], Tilburg Pregnancy Distress Scale [45], and Pregnancy-Related Anxiety Scale [38, 39], were all validated for use with participants in at least one low- or middle-income country [49–53].

There are many measures of pregnancy-related anxiety which were implemented in low- or middle-income countries, but most measures were not validated for use in those contexts before implementation. Furthermore, most papers provided limited information about the translation or adaptation processes undertaken before administering measure in a new language or context. Indeed, in many papers we had difficulty even identifying what language the measure was implemented in or if any changes had been made to it in order to make it locally relevant. While information on the reliability of pregnancy-related anxiety measures was included in most (65.4%) of the included studies, evidence of validity of the measures was less common (with any evidence included in only 45.4% of studies). Few of the studies mentioned this validity issue when discussing the limitations of their research. As shown in the COSMIN assessment, none of the validation studies used clinical interviews to determine cut-offs or the specificity / sensitivity of the measure (Supplemental Fig. 1), and many of the validation studies had substantial gaps in terms of face validity, predictive validity, and measurement invariance, among others.

## Discussion

This systematic review is the first to examine how pregnancy-related anxiety is being assessed in research worldwide. We found that there are nearly twenty tools which are used with some frequency, although just three of the measures (PRAQ, PRAS, CWS) are used in more than half of the papers. Research in this area is growing, with an increasing number of papers published on pregnancy-related anxiety each year, and studies conducted in 33 countries so far. Pregnancy-related anxiety is still predominantly assessed in high-income countries, in line with a scarcity of research on mental health in low- and middle-income countries generally [54–56].

Despite pregnancy-related anxiety being relatively frequently assessed in low- and middle-income countries, only two measures were originally developed for use in this context, and only four measures have been validated for use in any low- or middle-income country population (PRAQ, PRAS, CWS, and Tilburg Pregnancy Distress Scale. Although measures were used in Africa and South America, none of the measures were *validated* for use in any country in Africa, and only one was validated for use with a population in South America (in Brazil [53]). This is a substantial issue. The concept analysis by Bayrampour [5] found that underlying aspects of pregnancy-related anxiety differed by cultural and economic context, with women in low- and middle-income countries identifying different aspects of pregnancy-related anxiety that are salient to them than women in high-income countries. Measures are being applied in contexts where they have not been validated, and these measures may therefore fail to capture locally relevant or culturally specific aspects of pregnancy-related anxiety. The use of valid and reliable tools which incorporate all relevant aspects of pregnancy-related anxiety is necessary to ensure accurate quantification of pregnancy-related anxiety and to allow for the designing and testing of interventions to reduce pregnancy-related anxiety among women. This suggests that extant surveys may not be capturing locally relevant aspects of pregnancy-related anxiety in the countries in which they are implemented. This is problematic for the identification of predictors of pregnancy-related anxiety and for the development, evaluation, and implementation of appropriate interventions [54, 57]. It could result in falsely increased or decreased prevalence levels and may risk pathologizing groups based on mismeasurement.

We included all studies which said that they assessed pregnancy-related anxiety, regardless of whether the assessments were designed to test what we consider to be the construct of pregnancy-related anxiety. We did this because we wanted to understand what tools are being used when researchers want to assess pregnancy-related anxiety. In some cases, the tools being used to measure pregnancy-related anxiety do not seem to assess many of the important components of this construct. For example, the Cambridge Worry Scale includes some items which are specifically pregnancy- or birth-related, but others are more ‘general’ worries about housing, legal, financial, etc. troubles. When studying pregnancy-related anxiety, it is important that researchers use measures which capture all locally relevant components of this construct [5, 17, 58, 59].

This review was limited in four primary ways. First, this area of study is growing rapidly, but we conducted our initial search in August 2020, and so will have missed recent work. Second, because of the way that we used Rayyan for the title/abstract screening, we are unable to provide inter-rater reliability of the reviewers. Third, although we attempted to sample from a variety of databases including Africa Wide in order to access a wide range of papers, our review was limited by its focus solely on papers published in English and by the inaccessibility of full texts of some of the articles. Including only those papers published in English may have limited the number of studies included, biasing towards studies from English-speaking countries. Finally, multiple studies include measures with similar or the same names as other measures, and many do not cite the originating scale and provide few details of the measure used. This made identifying which measure was used very difficult for a handful of the included papers. We tried our best to identify the underlying measure used based on the description of the measure, number of items, and Likert scale, but may have miscategorized some.

## Conclusion

This systematic review of all studies which have assessed pregnancy-related anxiety highlights the variability in measurement, context, and study design. It suggests that pregnancy-related anxiety is being increasingly frequently assessed, but that this is often done using measures that were developed in very different contexts to which they are being implemented and may therefore have limited validity. There is an urgent need for researchers studying this important topic to focus on the quality of the measures being used, to validate measures before they are used in a new context or language, and to develop and validate new measures in low- and middle-income country contexts.

## Supplementary Information

**Additional file 1: Supplementary Figure 1.** COSMIN checklist for validation studies.

## Data Availability

All data analysed in this review are available in the Open Science Foundation repository (10.17605/OSF.IO/Z3VUX): https://osf.io/z3vux/.
