# Supplementary material for: Measurement of pregnancy-related anxiety worldwide: a systematic review
Source: BMC Pregnancy Childbirth. 2022 Apr 15;22:331. doi: 10.1186/s12884-022-04661-8 (PMC9013052; doi:10.1186/s12884-022-04661-8)
Supplement: Supplementary file 1 — Additional file 1: Supplementary Figure 1. COSMIN checklist for validation studies. [file 12884_2022_4661_MOESM1_ESM.docx]

*Supplementary Figure 1*. COSMIN checklist for validation studies.

|  | PrA tool | Construct clearly described | Development/translation/adaptation process provided | Description of items, subscales, and scoring | Participant eligibility criteria stated | Participants assessed comprehensibility of items | Experts assessed comprehensibility and comprehensiveness of items | Factor analysis conducted with an appropriate number of participants | Cronbach’s alpha reported for each subscale and the full scale | Cross-cultural validity/measurement invariance assessed | Evidence of intra- or inter-rater reliability | Evidence of convergent validity | Evidence of longitudinal validity or responsiveness of the tool |
| --- | --- | --- | --- | --- | --- | --- | --- | --- | --- | --- | --- | --- | --- |
| Derya et al. (2018) | PRAQ-R2 | X | X | X | X | X | X | X | X |  | X | X |  |
| Alderdice et al. (2011)* | PDQ | X |  |  | X |  |  | X | X |  |  |  |  |
| Anderson et al. (2019)* | PRAQ-R2 | X |  | X |  |  |  |  | X |  |  | X |  |
| Askarizadeh et al. (2017) | PRAQ-17 | X | X | X | X |  | X | X | X |  | X | X |  |
| Bayrampour et al. (2019) | PRAQ-R2 | X | X | X | X | X | X | X | X |  | X |  |  |
| Boerkhost et al (2020)* | TPDS | X |  | X | X |  |  | X | X |  | X |  |  |
| Brunton et al. (2018) | PrAS | X |  | X |  |  |  | X | X |  |  |  |  |
| Brunton et al. (2019) | PrAS | X | X | X |  |  | X | X | X |  |  | X |  |
| Chan et al. (2019) | PRAQ-R | X | X | X | X | X | X | X | X |  |  | X |  |
| Côté-Arsenault et al. (2003) | PAS (5 item) | X | X | X | X | X | X | X | X |  |  | X |  |
| Dellagiulia et al. (2019) | PRAQ-R2 | X | X | X |  | X | X | X |  | X |  | X |  |
| Fallon et al. (2016) | PSAS | X | X | X | X | X | X | X | X |  | X | X | X |
| Hirsch et al. (2017) | PDM | X | X | X | X |  | X | X | X |  |  | X |  |
| Huiznik et al (2016) | PRAQ-R2 | X | X | X | X |  |  | X | X | X |  |  |  |
| Huiznik et. (2004) | PRAQ-R* | X |  | X | X |  |  | X | X |  |  | X |  |
| Johnson and Slade (2002) | WDEQ | X |  | X | X |  |  | X | X |  |  | X |  |
| Kurt and Aslan (2020) | PrAS | X | X | X | X | X | X | X | X |  | X | X |  |
| Mortazavi and Akaberi (2016) | CWS | X | X | X | X | X | X | X | X |  |  | X |  |
| Mortazavi and Akaberi (2018) | ASP | X | X | X | X | X | X | X | X |  |  | X |  |
| Mudra et al. (2019) | PRAQ-R2 | X | X | X | X | X | X | X | X |  |  | X |  |
| Navidpour et al. (2015) | PWSQ | X | X | X | X |  | X | X | X |  | X | X |  |
| Ohman et al. (2003) | CWS | X | X | X |  |  |  |  | X |  |  |  |  |
| Olivia-Perez et al (2019) | PRT | X | X | X | X | X | X | X | X |  |  | X |  |
| Peterson et al. (2009) | CWS | X | X | X |  | X | X | X | X |  |  | X |  |
| Pitel at al. (2020) | WDEQ | X | X | X | X |  |  | X | X |  |  | X |  |
| Purkhajuee et al. (2016) | PRAQ-17 | X |  | X | X |  |  | X | X |  |  | X |  |
| Reiser at al. (2019) | FHAI | X | X | X | X | X |  | X | X |  |  | X |  |
| Vasquez et al. (2018) | PRAQ | X | X | X | X | X | X | X | X |  |  | X |  |
| Volpato et al (2019) | TPDS | X | X | X | X | X | X | X | X |  | X | X |  |

PrA = pregnancy-related anxiety, PDQ = Prenatal distress questionnaire, TPDS = Tilburg Pregnancy Distress Scale, PSAS = Postpartum specific anxiety scale, PDM = Prenatal distress measure, ASP = Anxiety scale for pregnancy PWSQ = Pregnancy’s Worries and Stress Questionnaire, PRT = Pregnancy-Related Thoughts Scale, FHAI = Fetal Health Anxiety Inventory

* Although includes a validation of the measure, tool development and/or translation is not the focus of the paper.
